# A prognostic survival model for women diagnosed with invasive breast cancer in Queensland, Australia

**DOI:** 10.1007/s10549-022-06682-5

**Published:** 2022-07-28

**Authors:** Peter D Baade, Helen Fowler, Kou Kou, Jeff Dunn, Suzanne K Chambers, Chris Pyke, Joanne F Aitken

**Affiliations:** 1 Cancer Council Queensland, Brisbane, Australia; 2 School of Mathematical Sciences, Queensland University of Technology, Brisbane, Australia; 3 Menzies Health Institute Queensland, Griffith University, Gold Coast, Australia; 4 Prostate Cancer Foundation of Australia, Sydney, Australia; 5 Faculty of Health Sciences, Australian Catholic University, Sydney, Australia; 6 Mater Hospitals South Brisbane, Brisbane, Australia; 7 School of Public Health and Social Work, Queensland University of Technology, Brisbane, Australia; 8 School of Public Health, The University of Queensland, Brisbane, Australia

**Keywords:** Breast cancer, Survival, Prognosis, Stage, Screening, Australia

## Abstract

**Purpose:**

Prognostic models can help inform patients on the future course of their cancer and assist the decision making of clinicians and patients in respect to management and treatment of the cancer. In contrast to previous studies considering survival following treatment, this study aimed to develop a prognostic model to quantify breast cancer-specific survival at the time of diagnosis.

**Methods:**

A large (*n* = 3323), population-based prospective cohort of women were diagnosed with invasive breast cancer in Queensland, Australia between 2010 and 2013, and followed up to December 2018. Data were collected through a validated semi-structured telephone interview and a self-administered questionnaire, along with data linkage to the Queensland Cancer Register and additional extraction from medical records. Flexible parametric survival models, with multiple imputation to deal with missing data, were used.

**Results:**

Key factors identified as being predictive of poorer survival included more advanced stage at diagnosis, higher tumour grade, “triple negative” breast cancers, and being symptom-detected rather than screen detected. The Harrell’s C-statistic for the final predictive model was 0.84 (95% CI 0.82, 0.87), while the area under the ROC curve for 5-year mortality was 0.87. The final model explained about 36% of the variation in survival, with stage at diagnosis alone explaining 26% of the variation.

**Conclusions:**

In addition to confirming the prognostic importance of stage, grade and clinical subtype, these results highlighted the independent survival benefit of breast cancers diagnosed through screening, although lead and length time bias should be considered. Understanding what additional factors contribute to the substantial unexplained variation in survival outcomes remains an important objective.

**Supplementary Information:**

The online version contains supplementary material available at 10.1007/s10549-022-06682-5.

## Background

Breast cancer is one of the most common cancers diagnosed in women [1]. It is a highly heterogenous disease with varying prognosis [2]. Prognostic models can help inform patients on the future course of their cancer and assist the decision making of clinicians and patients in respect to management and treatment of the cancer. Such models have been developed and validated to predict breast cancer survival among specific subgroups of patients, commonly based upon clinical and pathological prognostic factors. For example, the Nottingham Prognostic Index (NPI) [3] predicts survival for patients with operable breast cancer based upon tumour size, lymph-node status and histological grade. Alternatively, the ‘PREDICT’ model [4]—and the more recent iteration ‘PREDICT v2.0’ [5]—predict survival among breast cancer patients who have undergone surgery, based upon patient age, pathological factors, treatment factors and mode of detection.

In a broader context, there is evidence to suggest that poor survival prognosis following a breast cancer diagnosis may be associated with a variety of factors, including but not limited to younger or older age [6, 7], socioeconomic disadvantage [8, 9], and patient comorbidity [10, 11]. In contrast to many previous studies looking at survival following treatment [12], the aim of this study was to develop a prognostic model to quantify breast cancer-specific survival at the time of diagnosis, using a range of information from the Breast Cancer Outcomes Study (BCOS), a large, prospective cohort of women diagnosed with invasive breast cancer in Queensland, Australia.

## Methods

### Data

The BCOS is a longitudinal study of women aged 20 to 79 years and diagnosed with invasive breast cancer in Queensland, Australia between 31st March 2010 and 30th June 2013. Eligibility criteria for the study included the ability to speak and understand English and be without any cognitive impairment that would prevent participation in a phone interview.

A total of 5426 potentially eligible women were identified from the Queensland Cancer Register for inclusion in the study. Of these, doctor’s consent to contact was not provided for 688 women and 66 women were deceased. Of the 4672 women remaining, 3326 (71%) completed the interview and all but 3 (*n* = 3323) consented to their data from the study being prospectively linked with Registry data for follow-up purposes.

Full details of data collection procedures have been described previously [13, 14]. In summary, a validated, semi-structured telephone interview administered by trained health interviewers was used to obtain information relating to each woman’s experience of the pathway to breast cancer diagnosis. A self-administered questionnaire was completed by each woman following their telephone interview in which information was collected about detection of breast cancer (for example, information on frequency of screening and presence of symptoms), health status (including information on the presence of other chronic conditions or comorbidities, Body Mass Index (BMI) and physical activity levels) and socio-demographic characteristics (including age, education, marital status, employment, income, and area of residence).

Additional data including tumour characteristics were obtained by linkage of BCOS data with the Queensland Cancer Register. Additional clinical information was sourced from medical records. The linkage with the Queensland Cancer Register also provided information on all deaths that had occurred up to 31^st^ December 2018, and the cause of death, which was classified according to the coding of the International Statistical Classification of Diseases and Related Health Problems, tenth revision (ICD10) [15].

The medical records provided a composite variable of stage at diagnosis in addition to information on the individual staging components of tumour size and nodes involvement. A variable indicating clinical subtype of the tumour was created based upon the positive or negative status of oestrogen receptor (ER), progesterone receptor (PR) and human epidermal growth factor receptor 2 (HER2), using the St Gallen 2013 criteria[16] excluding Ki67, as has been done elsewhere [6]. Subtypes were Luminal A-like (ER+PR+HER2−), Luminal B-like HER2-negative (ER+PR−HER2−), Luminal B-like HER2-positive (ER+PR+HER2+or ER+PR−HER2+), HER2-positive (ER−PR−HER2+) or Triple Negative (ER−PR−HER2−). Where any of ER, PR or HER2 were missing (*n* = 675, 20.3%), or in combination of ER-PR+and HER2± (*n* = 23, 0.7%), subtype was treated as missing or considered uncertain subtype and set to missing.

### Outcome

Breast cancer-specific survival was the outcome of interest. Death due to breast cancer was defined by an ICD10-code of C50 for cause of death.

### Analysis

Approximately forty of the available patient, tumour, clinical, and healthcare variables were considered as possible variables of interest, based on a priori knowledge or evidence presented within the scientific literature of associations with cancer survival.

Cross tabulations examined the distribution of variables against the outcome variable of breast cancer-specific death. Univariate analyses using Cox Proportional Hazards models and stratified Kaplan–Meier survival estimates were performed to check for evidence of associations between variables of interest and breast cancer-specific death. Variables showing an unadjusted association with the outcome were then considered for inclusion in the prognostic model.

Determining the final prognostic model was done in several steps. Multiple imputation by chained equations was initially used to deal with very small percentages of missing values in stage at diagnosis (1.7% missing), tumour size (2.2%) and tumour grade (1.4%) variables, and in the clinical subtype variable, in which 20% of values were missing—assumed missing at random [17]. Based upon standard practice [17], twenty five imputations were performed on the basis that approximately 22% of all patients had at least one of the values missing for these variables.

Following imputation, flexible parametric survival models [18] with multivariate fractional polynomials (MFP) [19] were used to guide the selection of variables to include in the final model. This process employed a backward selection approach, sequentially removing the least significant variable from the model until all remaining variables in the model reported *p* values < 0.05. We ran analyses forcing age at diagnosis to be retained in the model on a priori grounds and given its practicality in a prognostic tool, and a separate analysis without this constraint. We then used flexible parametric models to determine whether the covariates retained in the model had a time-varying effect on the outcome.

The best fitting model in respect to the scale and number of degrees of freedom of the baseline spline function was determined using the Bayes Information Criterion (BIC) statistic [20]. To assess the discrimination and explained variation of the final prognostic model, and the individual contribution of each covariate in the model, we used the D statistic and associated *R*^2^_D_ proposed by Royston and Sauerbrei [21]. The measure of discrimination of a prognostic model indicates how well the model is able to differentiate between patient outcomes. We calculated the area under the Receiver-Operating Characteristic (ROC) curves for 5-year breast cancer-specific mortality, to ascertain how well the model identified patients with poorer survival prognosis. Additionally, we estimated the Harrell’s C-index [22], a measure of goodness-of-fit, for the final prognostic model and models singularly including each covariate. When deriving the prognostic model, we included tumour staging as a potential prognostic factor using the composite staging variable in the first instance, as that included information on the presence of metastases. We ran separate analyses using the tumour and nodes staging components to compare the discrimination, explained variation, and goodness of fit of both final models.

Post-estimation prediction was used to estimate the survival probability up to 5 years following breast cancer diagnosis, based upon differing combinations of the prognostic factors. All analyses were conducted using Stata 16 [23].

## Results

Of the 3323 women who were part of this study, 251 (7.6%) had died by the time of last follow-up, and the cause of 174 (69%) of these deaths was attributed to breast cancer. The median follow-up time of the study was 6.8 years (range 0.9–8.8 years).

Nine variables showed evidence of an association with breast cancer-specific survival based upon univariate analyses (Table 1). The three staging variables (the composite variable and individual tumour and nodes staging components) were associated with the outcome. Other variables associated with breast cancer-specific survival were age at diagnosis, mode of detection (i.e., via screening or symptoms), tumour grade, clinical subtype, diagnostic interval (time between symptoms or screening occurring and receiving a cancer diagnosis) and private health insurance status. Other variables considered are listed in Supplementary Table 1.

**Table 1:** Variables associated with breast cancer-specific survival

	All patients	Died due to breast cancer		*p* value*
	Total (*N* = 3323)	No (*n* = 3149)	Yes (*n* = 174)	
	*N*(%^a^)	*n*(%)	*n*(%)	
Age at diagnosis (years)				< 0.01
< 40	187 (5.6)	168 (89.8)	19 (10.2)	
40–49	710 (21.4)	681 (95.9)	29 (4.1)	
50–69	1970 (59.3)	1876 (95.2)	94 (4.8)	
70+	456 (13.7)	424 (93.0)	32 (7.0)	
Stage at diagnosis				< 0.01
Stage I	1600 (48.1)	1580 (98.8)	20 (1.3)	
Stage IIA/IIB	1284 (38.6)	1221 (95.1)	63 (4.9)	
Stage IIIA/IIIB/IV	381 (11.5)	298 (78.2)	83 (21.8)	
Missing	58 (1.7)	50 (86.2)	8 (13.8)	
Tumour size				< 0.01
< 15 mm	1358 (40.9)	1345 (99.0)	13 (1.0)	
15–29 mm	1214 (36.5)	1151 (94.8)	63 (5.2)	
30 mm+	678 (20.4)	595 (87.8)	83 (12.2)	
Missing	73 (2.2)	58 (79.5)	15 (20.5)	
Positive lymph nodes				< 0.01
No	2182 (65.7)	2124 (97.3)	58 (2.7)	
Yes	1141 (34.3)	1025 (89.8)	116 (10.2)	
Tumour grade				< 0.01
Low	619 (18.6)	612 (98.9)	7 (1.1)	
Intermediate	1585 (47.7)	1535 (96.8)	50 (3.2)	
High	1074 (32.3)	963 (89.7)	111 (10.3)	
Missing	45 (1.4)	39 (86.7)	6 (13.3)	
Clinical subtype				< 0.01
Luminal A	1805 (54.3)	1748 (96.8)	57 (3.2)	
Luminal B (HER2 −ve)	187 (5.6)	173 (92.5)	14 (7.5)	
Luminal B (HER2 + ve)	265 (8.0)	251 (94.7)	14 (5.3)	
HER2 positive	129 (3.9)	116 (89.9)	13 (10.1)	
Triple negative	262 (7.9)	229 (87.4)	33 (12.6)	
Missing	675 (20.3)	632 (93.6)	43 (6.4)	
Mode of detection				< 0.01
Symptoms	1681 (50.6)	1534 (91.3)	147 (8.7)	
Screening	1642 (49.4)	1615 (98.4)	27 (1.6)	
Diagnostic delay				< 0.01
Less than 30 days	2309 (69.5)	2206 (95.5)	103 (4.5)	
30–59 days	518 (15.6)	491 (94.8)	27 (5.2)	
60+ days	496 (14.9)	452 (91.1)	44 (8.9)	
Private health insurance				0.03
None	871 (26.2)	811 (93.1)	60 (6.9)	
DVA/some insurance	342 (10.3)	326 (95.3)	16 (4.7)	
Full insurance	2110 (63.5)	2012 (95.4)	98 (4.6)	

*DVA* Department of Veteran Affairs; *HER2* human epidermal growth factor receptor 2

*p values of Likelihood Ratio X2 Statistic from Univariate Cox Regression

^a^Percentage of all patients

Around half of the patients (48%) who had died from their breast cancer had a stage III or stage IV diagnosis, while approximately three-quarters (72%) of the patients who died were aged 50 years or more (Table 1). The majority (85%) of the patients who died had a symptomatic diagnosis of breast cancer. Over half (54%) of the patients had Luminal A subtype, while approximately 3% of the patients with this subtype died due to their breast cancer. By contrast, far fewer patients (8%) had a tumour of Triple Negative subtype, but 13% of these patients died from their breast cancer. Almost two-thirds (64%) of patients who died due to their breast cancer had tumours with high grade (poorly differentiated) cells. A quarter (25%) of patients who died had a diagnostic interval of 60 or more days. Over half of the patients who died (56%) had full health insurance.

### Prognostic model

The final model contained age, stage at diagnosis (composite staging information), tumour grade, clinical subtype, and mode of detection. Age was modelled using restricted cubic splines with two internal knots and centred at age 60 (approximate to the mean age of patients: 57.5 years). The model with the baseline spline function on the probit scale with 2 degrees of freedom (df) had the lowest BIC statistic. From the analysis using flexible parametric models, there was no evidence of any covariates having a time-varying effect on the outcome.

**Fig. 1:**
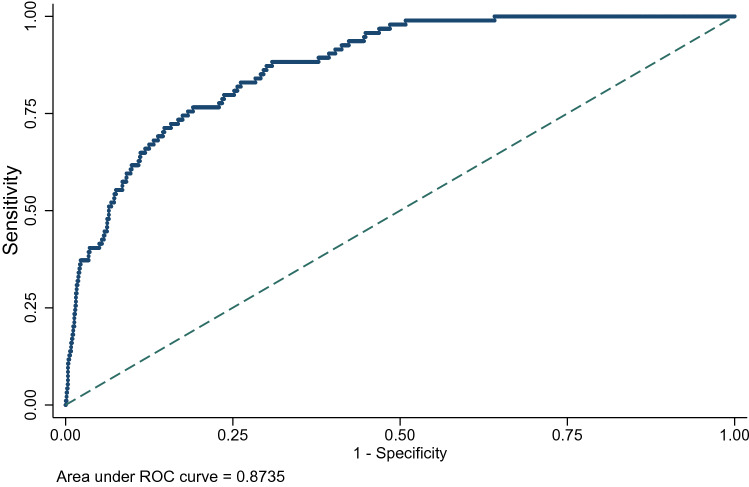
Receiver-Operating Characteristic (ROC) curve for five-year breast cancer-specific mortality

The measures of discrimination (D statistic) and explained variation (*R*^2^_D_), and Harrell’s C goodness-of-fit statistic of the final prognostic model and each covariate in the model is provided in Table 2. The model explained over one-third of variation in survival (*R*^2^_D_ 0.36; 95% CI 0.30, 0.42), with a D statistic of 1.20. The Harrell’s C-statistic was 0.84 (95% CI 0.82, 0.87), a value over 0.80 indicating a strong predictive model. This model with the composite staging variable compared more favorably to the model with tumour size and nodes involvement included separately (*R*^2^_D_ 0.32; 0.26, 0.39; D-statistic 1.11 and Harrell’s C-statistic 0.83; 0.80, 0.85, results not shown). Additionally, the area under the ROC curve for 5-year mortality (Fig. 1) was 0.8735. Being close to 1, this value suggests the model performs well in distinguishing between patient outcomes.

**Table 2:** Prognostic model discrimination (D-statistic) and explained variation (R2D statistic)

	D-statistic (95% CI)	R2D (95% CI)	Harrell’s C-statistic (95% CI)
Prognostic model	1.20 (1.04, 1.36)	0.36 (0.30, 0.42)	0.84 (0.82,0.87)
Singularly removing listed variable from the prognostic model			
Age at diagnosis	1.17 (1.01, 1.33)	0.35 (0.29,0.41)	0.84 (0.81, 0.86)
Clinical subtype	1.15 (0.99, 1.30)	0.34 (0.28, 0.40)	0.84 (0.81, 0.86)
Tumour grade	1.12 (0.97, 1.28)	0.33 (0.27, 0.39)	0.83 (0.80, 0.86)
Mode of detection	1.11 (0.96, 1.27)	0.33 (0.27, 0.39)	0.83 (0.80, 0.86)
Stage at diagnosis	0.88 (0.74, 1.02)	0.23 (0.17, 0.29)	0.79 (0.76, 0.82)
Singularly including listed variable in a model			
Age at diagnosis	0.09 (0.19, − 0.19)	< 0.01*	0.54 (0.49, 0.58)
Clinical subtype	0.51 (0.37, 0.65)	0.09 (0.05, 0.14)	0.64 (0.60, 0.69)
Tumour grade	0.70 (0.55, 0.85)	0.16 (0.10, 0.22)	0.69 (0.66, 0.73)
Mode of detection	0.79 (0.61, 0.95)	0.19 (0.13, 0.26)	0.68 (0.65, 0.71)
Stage at diagnosis	0.94 (0.79, 1.08)	0.26 (0.20, 0.32)	0.77 (0.73, 0.81)
Including listed variable in a model with stage at diagnosis			
Age at diagnosis	0.84 (0.70, 0.98)	0.23 (0.16, 0.27)	0.79 (0.75, 0.82)
Clinical subtype	0.99 (0.84, 1.14)	0.28 (0.22, 0.34)	0.80 (0.77, 0.84)
Tumour grade	1.06 (0.91, 1.22)	0.31 (0.25, 0.37)	0.81 (0.78, 0.84)
Mode of detection	1.02 (0.87, 1.18)	0.29 (0.23, 0.35)	0.80 (0.77, 0.83)

*CI* confidence interval

*Confidence intervals not reported due to low value of R2D

Stage at diagnosis was the variable offering the largest contribution to the final model: stage alone explained approximately 26% (*R*^2^_D_ 0.26; 0.20, 0.32) of the variation in survival. Including tumour grade or mode of detection separately in a model with stage explained a further 5% or 3% of variation, respectively (Table 2). Conversely, a model including all covariates in the prognostic model except stage explained approximately 23% of the variation in survival (*R*^2^_D_ 0.23; 0.17, 0.29). This similarity to that of the model with stage alone suggested the existence of correlation between covariates.

Clinical subtype and age at diagnosis appeared to offer a very small amount of discrimination and explained variation in the survival model. Individually removing clinical subtype or age from the final model led to a reduction in D of 0.05 and 0.03 and a reduction in *R*^2^_D_ of 0.02 and 0.01, respectively. When age was included alone in a model it explained less than 1% of the variation in survival.

### Predicted survival probabilities

**Fig. 2:**
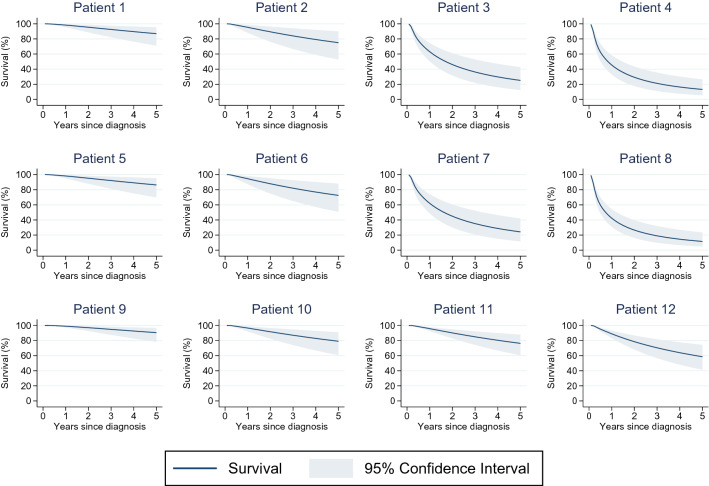
Predictors breast cancer-specific survival for 12 hypothetical patients. Details of 12 patients described in Table 3

The model was used to predict breast cancer-specific survival, that is, survival under an assumption that breast cancer is the only possible cause of death. To demonstrate the application of this model, we predicted 1-year and 5-year survival probabilities of 12 hypothetical patients, using different scenarios of prognostic factor combinations to characterise these patients (Table 3, Fig. 2). Generally, predicted survival was poorer among patients with advanced stage of disease and of older age. Survival tended to be higher among patients whose breast cancer was detected via screening rather than from symptoms and who had tumours of early stage and low grade. Patients with tumours of Luminal A like clinical subtype had a better prognosis, while those with Triple Negative subtype tended to fare the worst.

**Table 3:** Predicted 1-year and 5-year breast cancer-specific survival of twelve hypothetical patients, according to values of prognostic factors

Patient number	Age at diagnosis (years)	Stage at diagnosis	Mode of detection	Grade	Clinical subtype^a^	Predicted breast cancer-specific survival (95% CI)	
						One-year survival (%)	Five-year survival (%)
1	35	1	Symptoms	Low	Luminal A like	98.3 (95.4, 99.5)	87.0 (71.1, 95.5)
2	35	1	Symptoms	Low	Triple negative	95.4 (88.2, 98.5)	75.1 (52.7, 90.1)
3	35	3 or 4	Symptoms	High	Luminal A like	63.1 (50.4, 74.5)	25.1 (12.4, 42.6)
4	35	3 or 4	Symptoms	High	Triple negative	45.5 (33.0, 58.5)	13.2 (5.4, 26.4)
5	55	1	Screening	Low	Triple negative	98.2 (95.0, 99.5)	86.3 (69.9, 95.3)
6	55	1	Symptoms	Low	Triple negative	94.6 (87.3, 98.1)	72.5 (50.7, 88.1)
7	55	3 or 4	Screening	High	Triple negative	62.0 (48.6, 74.1)	24.2 (11.6, 42.0)
8	55	3 or 4	Symptoms	High	Triple negative	42.4 (31.0, 54.4)	11.6 (4.7, 23.4)
9	75	1	Screening	Low	Luminal A like	99.0 (97.2, 99.7)	90.4 (78.2, 96.7)
10	75	1	Symptoms	Low	Luminal A like	96.5 (92.0, 98.7)	79.0 (60.7, 91.1)
11	75	2	Screening	Intermediate	Luminal A like	95.7 (92.5, 97.7)	76.3 (60.5, 87.7)
12	75	2	Symptoms	Intermediate	Luminal A like	88.9 (83.2, 93.1)	58.6 (41.4, 74.3)

*CI* confidence interval; *ER* oestrogen receptor; *HER2* human epidermal growth factor receptor 2; *PR* progesterone receptor

^a^Luminal A like: ER+PR+HER2−; Triple negative: ER−PR−HER2−

For example, a 35-year-old symptomatic patient with stage 1 disease with tumour of low grade and Luminal A-like clinical subtype (Patient 1) had a predicted 5-year survival of 87.0% (95% CI 71.1%, 95.5%), while a patient with the same characteristics except for a tumour of Triple Negative subtype (Patient 2) had a predicted survival of 75.1% (52.7%, 90.1%). A screen-detected 55-year-old patient with stage 1 tumour of low grade and Triple Negative subtype (Patient 5) had a predicted survival of 86.3% (69.9%, 95.3%), while a symptomatic 55-year-old patient with advanced stage (stage 3 or 4) breast cancer of high grade and Triple Negative subtype (Patient 8) had a predicted 5-year survival of 11.6% (4.7%, 23.4%). A 75-year-old screen-detected patient with a stage 2 diagnosis, intermediate tumor grade and Luminal A-like subtype (Patient 11) had a predicted 5-year survival of 76.3% (60.5%, 87.7%) while a symptomatic patient of the same age and with the same tumour characteristics (Patient 12) had a poorer predicted survival of 58.6% (41.4%, 74.3%).

## Discussion

This study used detailed data from a longitudinal, population-based cohort of breast cancer patients, supplemented with cancer registry data. This enabled a greater range of potential prognostic factors to be considered in the development of a prognostic survival model than would be possible using cancer registry data alone. Importantly, our results confirmed the prognostic importance of stage at diagnosis, tumour grade, and clinical subtype for women diagnosed with breast cancer, along with age at diagnosis, but also highlighted the survival benefit of breast cancers diagnosed through screening, independently of the measured clinical characteristics.

To our knowledge, this is the first prognostic model of breast cancer survival that has been developed using flexible parametric survival models. Cox Proportional Hazards models have more typically been used for this purpose [24]. The Cox models follow an assumption that each covariate has a constant impact on the hazard throughout the follow-up period [25]. Using flexible parametric models, we were able to evaluate potential time-dependent effects of covariates while developing the final prognostic model. An additional benefit with using this approach was the ability to predict survival at different time points following breast cancer diagnosis, according to different combinations of prognostic factors.

The clinical benefit and common use of prognostic breast cancer survival models has been demonstrated through previous studies. The Nottingham Prognostic Index (NPI) allocates patients into groups based on their survival prognosis and predicts 5-year survival. It has performed well under validation using independent datasets [26, 27], and has been used widely in breast cancer management, for example, in selection for breast conserving surgery or for adjuvant therapy [3]. Similarly, the ‘PREDICT’ prognostic tool[4] is intended for use by patients and clinicians to assess the impact of different treatments on survival following surgery. Since being released as an online tool in 2011 it has been accessed frequently, and from locations around the world [5].

The NPI was constructed using data of approximately 400 breast cancer patients treated by mastectomy at a hospital in Nottingham, England, and is based upon three factors (tumour size, grade, and lymph-node stage). Like the NPI, our prognostic model offers simplicity, an important criterion when developing a clinically useful model [28]. Stage at diagnosis was the strongest predictor in our prognostic model. The staging components of tumour size and nodal status are the two most common predictors in published breast cancer survival models [24].

The prognostic model highlighted survival differences between women whose breast cancer was screen-detected and women whose breast cancer was detected via symptoms. These differences may be influenced by lead time [29], where screening has brought the diagnosis forward and represents an artificial addition to the survival time of screen-detected patients. There is also a possibility of length bias [29], where screening has detected slow-growing breast cancers while they are screen-detectable but not yet symptomatic. The prognostic model predicted survival differences even after adjustment for stage at diagnosis, thus after accounting for the possibility of earlier stage detection among screened women. Findings such as these could be useful in a public health setting—for example, in health promotion initiatives highlighting benefits of breast cancer screening. In Australia, women aged between 50 and 74 years are invited for free breast cancer screening every two years [30], while women aged 40–49 years and those aged over 74 years are also eligible for free screening. Our prognostic model predicted better survival among BCOS patients with a screen-detected versus symptomatic breast cancer diagnosis, regardless of age, and clinical characteristics.

Potential data limitations include the possibility of selection bias, as the study cohort excluded women with more advanced stage of disease who were too unwell or unable to take part [13]. As a consequence of this, the prognostic model may overestimate survival among women with advanced stage of disease, relative to what may be seen among a more representative population of breast cancer patients. The exclusion of patients with advanced stage may also lead to an underrepresentation of patients with characteristics traditionally associated with advanced stage cancer and poorer prognosis. For example, socio-economically disadvantaged breast cancer patients tend to have greater risk of presenting with advanced stage of disease [31, 32], and tend to have poorer survival outcomes [8, 33, 34]. With the exception of mode of detection, the prognostic model contained variables sourced from the Queensland Cancer Registry or from medical records. Mode of detection information was collected from BCOS patients using computer-assisted telephone interview, by asking how the breast cancer was first detected. Self-reporting biases may arise during data collection via survey questionnaire methods [35]. The composite stage at diagnosis variable used in the prognostic model was obtained from review of medical records. There was a small amount of missing information within the stage at diagnosis variable and within some of the other variables included in the prognostic model. We used multiple imputation to handle this missing information, under an assumption the data were missing at random [36].

Externally validating prognostic models using independent datasets gives an indication of the predictive ability and generalisability of these models [28]. Some breast cancer prognostic survival models have shown weaker performance in independent populations [24]. Although our model has not yet been validated using an external dataset, internal validation of the model using Harrell’s concordance statistic indicated it had good ability to predict survival. The methodology used to develop our prognostic model has been used in the development of a prognostic survival model of invasive cutaneous melanoma [37]. The melanoma model offered greater discrimination than the breast cancer model (D-statistic of 1.53 versus 1.20), possibly due to the higher prognostic contribution that melanoma thickness had on melanoma survival compared to tumour diameter for breast cancer survival, even though both were the most important contributors in their respective prognostic model. Consistent with this, the prognostic factors in the melanoma model explained approximately half of the variation in survival while the breast cancer model explained one-third of the variation in survival.

Consistent with prognostic models for other cancer types [37, 38], this study has specifically focused on survival outcomes using information available at the time of diagnosis. While providing greater clarity about the role that socio-demographic, diagnostic and other clinical characteristics has on patient survival, there is an increasing role for tailored treatment plans for cancer patients that also consider additional information, including information about specific treatments, that become available during the management pathway [12]. As such, while our current model is novel in focusing on diagnostic-related variables, there is the potential to create an expanded prognostic model for this breast cancer cohort that incorporates additional post-diagnosis variables, including treatment-related factors, to compare with other published decision tools.

In conclusion, by using a large, population-based survey of Australian women diagnosed with breast cancer, this study provided novel insights into the important factors known at the time of diagnosis that influence survival outcomes that could not have been obtained through more typical cancer registry-based analyses. Assessing the performance of our model using other breast cancer cohorts would be a beneficial and informative next step. Given the large percentage of survival variation that was unexplained by this model, gaining a better understanding of what additional factors explain survival outcomes among women diagnosed with breast cancer will require dedicated research studies that include more comprehensive range of factors and/or more nuanced measurements.

## Supplementary Information

Below is the link to the electronic supplementary material.

Supplementary file1 (DOCX 20 kb)

## Data Availability

The de-identified dataset analysed during the current study is not publicly available due to the requirements of the original ethical approval but would be available from the corresponding author on reasonable request following a formal data sharing agreement.
