# Supplementary material for: A prognostic survival model for women diagnosed with invasive breast cancer in Queensland, Australia
Source: Breast Cancer Res Treat. 2022 Jul 28;195(2):191–200. doi: 10.1007/s10549-022-06682-5 (PMC9374611; doi:10.1007/s10549-022-06682-5)
Supplement: Supplementary file 1 — Supplementary file1 (DOCX 20 kb) [file 10549_2022_6682_MOESM1_ESM.docx]

Supplementary Table 1: Variables not associated with breast-cancer specific survival

|  |  | Died due to breast cancer | |  |
| --- | --- | --- | --- | --- |
|  | Total (N=3323) | No (n=3149) | Yes (n=174) | P-value* |
|  | N(%^a^) | n(%) | n(%) |  |
| **Patient characteristics** |  |  |  |  |
| *Education level* |  |  |  | 0.67 |
| High school or less | 1293 (38.9) | 1230 (95.1) | 63 (4.9) |  |
| Certificate / Vocational | 797 (24.0) | 757 (95.0) | 40 (5.0) |  |
| Diploma / Bachelors / Higher | 1231 (37.0) | 1160 (94.2) | 71 (5.8) |  |
| Missing | 2 (0.1) | 2 (100.0) | 0(0) |  |
| *Household income pre-diagnosis* |  |  |  | 0.46 |
| $0-$51,999 | 1216 (36.6) | 1152 (94.7) | 64 (5.3) |  |
| $52,000-$129,999 | 1271 (38.2) | 1206 (94.9) | 65 (5.1) |  |
| $130,000+ | 476 (14.3) | 455 (95.6) | 21 (4.4) |  |
| Missing | 3 (0.1) | 3 (100.0) | 0 (0) |  |
| *Marital status* |  |  |  | 0.74 |
| Married / living as married | 2438 (73.4) | 2312 (94.8) | 126 (5.2) |  |
| Not married / not living as married | 885 (26.6) | 837 (94.6) | 48 (5.4) |  |
| *Ethnic origin* |  |  |  | 0.06 |
| Caucasian / white | 3157 (95.0) | 2987 (94.6) | 170 (5.4) |  |
| Other | 126 (3.8) | 122 (96.8) | 4 (3.2) |  |
| Missing | 40 (1.2) | 40 (100.0) | 0 (0) |  |
| *Indigenous* |  |  |  | - |
| Yes | 53 (1.6) | 53 (100.0) | 0 (0) |  |
| No | 3270 (98.4) | 3096 (94.7) | 174 (5.3) |  |
| *Area level disadvantage* |  |  |  | 0.26 |
| Most disadvantaged | 574 (17.3) | 539 (93.9) | 35 (6.1) |  |
| Q2-Q4 | 2012 (60.5) | 1904 (94.6) | 108 (5.4) |  |
| Least disadvantaged | 737 (22.2) | 706 (95.8) | 31 (4.2) |  |
| *Accessibility and Remoteness Index of Australia* |  |  |  | 0.48 |
| Major city | 1838 (55.3) | 1749 (95.2) | 89 (4.8) |  |
| Inner regional | 793 (23.9) | 746 (94.1) | 47 (5.9) |  |
| Outer regional / remote / very remote | 692 (20.8) | 654 (94.5) | 38 (5.5) |  |
| **Tumour characteristics** |  |  |  |  |
| *Morphology* |  |  |  | 0.62 |
| Ductal types | 2553 (76.8) | 2420 (94.8) | 133 (5.2) |  |
| Lobular types | 551 (16.6) | 524 (95.1) | 27 (4.9) |  |
| Other types | 219 (6.6) | 205 (93.6) | 14 (6.4) |  |
| **Clinical characteristics** |  |  |  |  |
| *Number of comorbidities ^b^* |  |  |  | 0.29 |
| None | 740 (22.3) | 693 (93.6) | 47 (6.4) |  |
| One | 793 (23.9) | 757 (95.5) | 36 (4.5) |  |
| Two or more | 1790 (53.9) | 1699 (94.9) | 91 (5.1) |  |
| *Body Mass Index* |  |  |  | 0.76 |
| Underweight, <20 | 171 (5.1) | 161 (94.2) | 10 (5.8) |  |
| Healthy weight, 20-24 | 1121 (33.7) | 1063 (94.8) | 58 (5.2) |  |
| Overweight, 25-29 | 1043 (31.4) | 995 (95.4) | 48 (4.6) |  |
| Obese, 30+ | 972 (29.3) | 915 (94.1) | 57 (5.9) |  |
| Missing | 16 (0.5) | 15 (93.8) | 1 (6.3) |  |
| *History of heart attack* |  |  |  | 0.57 |
| No | 3251 (97.8) | 3081 (94.8) | 170 (5.2) |  |
| Yes | 64 (1.9) | 60 (93.8) | 4 (6.3) |  |
| Missing | 8 (0.2) | 8 (100.0) | 0 (0) |  |
| *History of angina pectoris* |  |  |  | 0.38 |
| No | 3220 (96.9) | 3049 (94.7) | 171 (5.3) |  |
| Yes | 92 (2.8) | 89 (96.7) | 3 (3.3) |  |
| Missing | 11 (0.3) | 11 (100.0) | 0 (0) |  |
| *History of hypertension* |  |  |  | 0.21 |
| No | 2179 (65.6) | 2073 (95.1) | 106 (4.9) |  |
| Yes | 1135 (34.2) | 1067 (94.0) | 68 (6.0) |  |
| Missing | 9 (0.3) | 9 (100.0) | 0 (0) |  |
| *History of high cholesterol* |  |  |  | 0.44 |
| No | 2355 (70.9) | 2227 (94.6) | 128 (5.4) |  |
| Yes | 958 (28.8) | 912 (95.2) | 46 (4.8) |  |
| Missing | 10 (0.3) | 10 (100.0) | 0 (0) |  |
| *History of stroke* |  |  |  | 0.56 |
| No | 3267 (98.3) | 3098 (94.8) | 169 (5.2) |  |
| Yes | 46 (1.4) | 42 (91.3) | 4 (8.7) |  |
| Missing | 10 (0.3) | 9 (90.0) | 1 (10.0) |  |
| *Diabetes* |  |  |  | 0.64 |
| No | 3063 (92.2) | 2904 (94.8) | 159 (5.2) |  |
| Yes | 255 (7.7) | 240 (94.1) | 15 (5.9) |  |
| Missing | 5 (0.2) | 5 (100.0) | 0 (0) |  |
| *Asthma* |  |  |  | 0.27 |
| No | 2727 (82.1) | 2580 (94.6) | 147 (5.4) |  |
| Yes | 591 (17.8) | 564 (95.4) | 27 (4.6) |  |
| Missing | 5 (0.2) | 5 (100.0) | 0 (0) |  |
| *Chronic bronchitis* |  |  |  | 0.33 |
| No | 3068 (92.3) | 2908 (94.8) | 160 (5.2) |  |
| Yes | 239 (7.2) | 225 (94.1) | 14 (5.9) |  |
| Missing | 16 (0.5) | 16 (100.0) | 0 (0) |  |
| *Emphysema* |  |  |  | 0.78 |
| No | 3275 (98.6) | 3103 (94.7) | 172 (5.3) |  |
| Yes | 44 (1.3) | 42 (95.5) | 2 (4.5) |  |
| Missing | 4 (0.1) | 4 (100.0) | 0 (0) |  |
| *History of stomach or duodenal ulcer* |  |  |  | 0.40 |
| No | 3115 (93.7) | 2952 (94.8) | 163 (5.2) |  |
| Yes | 192 (5.8) | 181 (94.3) | 11 (5.7) |  |
| Missing | 16 (0.5) | 16 (100.0) | 0 (0) |  |
| *History of migraines* |  |  |  | 0.25 |
| No | 2494 (75.1) | 2365 (94.8) | 129 (5.2) |  |
| Yes | 819 (24.6) | 774 (94.5) | 45 (5.5) |  |
| Missing | 10 (0.3) | 10 (100.0) | 0 (0) |  |
| *Osteoporosis* |  |  |  | 0.27 |
| No | 2960 (89.1) | 2803 (94.7) | 157 (5.3) |  |
| Yes | 340 (10.2) | 323 (95.0) | 17 (5.0) |  |
| Missing | 23 (0.7) | 23 (100.0) | 0 (0) |  |
| *Osteoarthritis* |  |  |  | 0.27 |
| No | 2505 (75.4) | 2377 (94.9) | 128 (5.1) |  |
| Yes | 775 (23.3) | 734 (94.7) | 41 (5.3) |  |
| Missing | 43 (1.3) | 38 (88.4) | 5 (11.6) |  |
| *Rheumatoid arthritis* |  |  |  | 0.65 |
| No | 3186 (95.9) | 3020 (94.8) | 166 (5.2) |  |
| Yes | 104 (3.1) | 97 (93.3) | 7 (6.7) |  |
| Missing | 33 (1.0) | 32 (97.0) | 1 (3.0) |  |
| *History of depression* |  |  |  | 0.44 |
| No | 2506 (75.4) | 2374 (94.7) | 132 (5.3) |  |
| Yes | 812 (24.4) | 770 (94.8) | 42 (5.2) |  |
| Missing | 5 (0.2) | 5 (100.0) | 0 (0) |  |
| *Been through menopause* |  |  |  | 0.56 |
| Yes | 2688 (80.9) | 2553 (95.0) | 135 (5.0) |  |
| No | 453 (13.6) | 427 (94.3) | 26 (5.7) |  |
| Don't know | 178 (5.4) | 165 (92.7) | 13 (7.3) |  |
| Missing | 4 (0.1) | 4 (100.0) | 0 (0) |  |
| *Years of HRT* |  |  |  | 0.20 |
| Not taken or <2 years | 2459 (74.0) | 2322 (94.4) | 137 (5.6) |  |
| 2-10 years | 555 (16.7) | 535 (96.4) | 20 (3.6) |  |
| >10 years | 302 (9.1) | 286 (94.7) | 16 (5.3) |  |
| Missing | 7 (0.2) | 6 (85.7) | 1 (14.3) |  |
| **Lifestyle characteristics** |  |  |  |  |
| *Smoking status* |  |  |  | 0.22 |
| Never smoked | 1884 (56.7) | 1794 (95.2) | 90 (4.8) |  |
| Used to smoke | 1168 (35.1) | 1104 (94.5) | 64 (5.5) |  |
| Current smoker | 266 (8.0) | 246 (92.5) | 20 (7.5) |  |
| Missing | 5 (0.2) | 5 (100.0) | 0 (0) |  |
| *Alcohol intake pre-diagnosis* |  |  |  | 0.27 |
| Less than once per month | 1288 (38.8) | 1215 (94.3) | 73 (5.7) |  |
| At least once per month | 2032 (61.1) | 1931 (95.0) | 101 (5.0) |  |
| Missing |  |  |  |  |
| *Physical activity levels* |  |  |  | 0.73 |
| Insufficient | 1443 (43.4) | 1371 (95.0) | 72 (5.0) |  |
| Sufficient | 1834 (55.2) | 1736 (94.7) | 98 (5.3) |  |
| Missing | 46 (1.4) | 42 (91.3) | 4 (8.7) |  |

* P-values of Likelihood Ratio X2 Statistic from Univariate Cox Regression

a: Percentage of all patients

b: Number of comorbidities was based on the following list: Heart attack; Angina; Hypertension; High cholesterol; Stroke; Diabetes; Asthma; Chronic bronchitis; Emphysema; Ulcer; Migraine; Osteoporosis; Osteoarthritis; Rheumatoid arthritis; Depression

Abbreviations: HRT, Hormone Replacement Therapy
